# Vitamin D Deficiency in Adult Patients with Schizophreniform and Autism Spectrum Syndromes: A One-Year Cohort Study at a German Tertiary Care Hospital

**DOI:** 10.3389/fpsyt.2016.00168

**Published:** 2016-10-06

**Authors:** Dominique Endres, Rick Dersch, Oliver Stich, Armin Buchwald, Evgeniy Perlov, Bernd Feige, Simon Maier, Andreas Riedel, Ludger Tebartz van Elst

**Affiliations:** ^1^Section of Experimental Neuropsychiatry, Department of Psychiatry and Psychotherapy, Medical Center – University of Freiburg, Freiburg, Germany; ^2^Department of Neurology, Medical Center – University of Freiburg, Freiburg, Germany; ^3^Institute for Clinical Chemistry and Laboratory Medicine, Medical Center – University of Freiburg, Freiburg, Germany

**Keywords:** schizophrenia, autism spectrum disorder, vitamin D, inflammation, mild encephalitis

## Abstract

**Introduction:**

Vitamin D has many immunomodulatory, anti-inflammatory, and neuroprotective functions, and previous studies have demonstrated an association between vitamin D deficiency and neuropsychiatric disease. The aim of our study was to analyze the prevalence of vitamin D deficiency in a 1-year cohort of adult inpatients with schizophreniform and autism spectrum syndromes in a naturalistic inpatient setting in Germany.

**Participants and methods:**

Our study was comprised of 60 adult schizophreniform and 23 adult high-functioning autism spectrum patients who were hospitalized between January and December of 2015. We compared our findings with a historical German reference cohort of 3,917 adults using Pearson’s two-sided chi-squared test. The laboratory measurements of 25-hydroxyvitamin D2/3 [25(OH)vitamin D] were obtained using a chemiluminescence immunoassay.

**Results:**

In the schizophreniform group, we found decreased (<20 ng/ml) 25(OH)vitamin D levels in 48/60 (80.0%) of the patients. In the autism spectrum group, decreased levels were detected in 18/23 (78.3%) of the patients. 25(OH)vitamin D deficiencies were found in 57.3% of the historical control group. Particularly, severe deficiencies (<10 ng/ml) occurred much more frequently in the schizophreniform (38.3%) and autism spectrum groups (52.2%), when compared to the control group (16.3%). The recommended 25(OH)vitamin D values of >30 ng/ml were observed in only 5% of the schizophreniform patients, 8.7% of the autism spectrum patients, and 21.9% of the healthy controls.

**Discussion:**

We found very high rates of 25(OH)vitamin D deficiencies in both patient groups and have discussed whether our findings might be related to alterations in the immunological mechanisms. Irrespective of the possible pathophysiological links between vitamin D deficiency and schizophrenia or autism spectrum disorders, a more frequent measurement of vitamin D levels seems to be justified in these patient groups. Further prospective, controlled, blinded, and randomized research should be conducted to analyze the effectiveness of vitamin D supplementation on the improvement of psychiatric symptoms.

## Introduction

The role of vitamin D levels in skeletal health is well known; for example, vitamin D deficiency can cause or exacerbate osteoporosis, lead to muscle weakness, and increase the risk of bone fractures (1). Moreover, vitamin D has many immunomodulatory, anti-inflammatory, and neuroprotective functions (2). Previous studies have demonstrated an association between vitamin D deficiency and metabolic (e.g., atherosclerosis), neoplastic (e.g., colon cancer), and immune disorders (e.g., Type 1 diabetes mellitus) (1, 3). In neuropsychiatric research, the associations between vitamin D deficiency and multiple sclerosis (MS), Parkinson’s disease, schizophreniform disorder, autism spectrum disorders, and Alzheimer’s disease have recently been described (4–6).

### Vitamin D Deficiency in Neuropsychiatry

The association between MS and vitamin D deficiency is an established issue in the respective neuropsychiatric research, and it may be an independent risk factor for the development of MS. Moreover, vitamin D seems to modulate the course of the disease, in that higher vitamin D levels are correlated with reduced MS activity (2, 7). Earlier studies have also shown clear indications of an association between vitamin D deficiency and schizophreniform disorder. In the largest study of psychoses to date, vitamin D deficiency was identified in 86% of the cases, and in 49%, a severe deficiency of <10 ng/ml was reported (8). A recent meta-analysis found vitamin D deficiencies in 65.3% of these patients (9). In another meta-analysis, statistically significantly lower vitamin D levels were found in autistic patients compared to healthy controls. However, most of these studies were performed in children (10). A developmental vitamin D deficiency might lead to alterations in the structural and functional (e.g., alterations in the dopaminergic functions) brain features. Furthermore, an adult deficiency may be associated with diverse immunological alterations, as well as neurochemical changes (6, 10).

### Vitamin D Metabolism

Vitamin D is produced in the skin through the conversion of provitamin D3 to previtamin D3; however, the nutritional intake of vitamin D2/3 is small. Vitamin D can be stored in fat tissues or modified in the liver by the 25-hydroxylase enzyme to 25-hydroxyvitamin D2/3, which is then transformed to the active metabolite 1,25-dihydroxyvitamin D2/3, mainly in the kidneys. The synthesis of vitamin D3 is enhanced by increased levels of parathyroid hormone, which in turn, increase the calcium concentrations. Reduced phosphate can also lead to the production of vitamin D3 (3). The serum level of 25-hydroxyvitamin D2/3 [in this paper abbreviated as “25(OH)vitamin D”] is an established marker of the current vitamin D status and was therefore measured in our study.

### Vitamin D Levels in Germany

The German Nutrition Society^1^ and the American Institute of Medicine^2^ have defined a vitamin D deficiency as levels <20 ng/ml. These references are based on calculations with respect to the distribution of the “vitamin D requirement curve” in the general population. Serum levels of ≥20 ng/ml are necessary for bone health in 97.5% of the individuals in the population (11); however, preferred vitamin D values are generally above 30 ng/ml (1, 3, 9, 12). A German reference cohort of adults is available from the Robert Koch Institute and was reported in a statement from the German Nutrition Society. In this cohort of 3,917 adult subjects ranging from 18 to 79 years, an average vitamin D level of 18 ng/ml was found, and overall vitamin D deficiency (levels <20 ng/ml) was found in 57.3% of subjects. The specific breakdown was as follows: 2% were below 5 ng/ml, 14.3% were 5–10 ng/ml, 41% were 10–20 ng/ml, and concentrations between 20 and 30 ng/ml were found in 20.8% of the subjects. Recommended vitamin D levels of >30 ng/ml were identified in 21.9% of these subjects (13).

### Rationale for the Study

In our clinic, we offer a broad diagnostic workup for patients with schizophreniform syndromes, including a broad range of laboratory measurements, cerebrospinal fluid (CSF) analyses, electroencephalography (EEG), and cerebral magnetic resonance imaging (cMRI) (14–16). In doing so, we have found non-specific immunological CSF alterations in 54.4% and overall abnormalities, including EEG and MRI findings, in 75.6% of the patients (14). Moreover, among our patients with autism spectrum disorders, we routinely perform laboratory analyses, EEGs, and cMRIs (17, 18). In our earlier structural imaging studies, we found no differences between those patients with high-functioning autism and the healthy controls (19), although neurochemical alterations in the glutamatergic prefrontal system were detected with MR spectroscopy (20, 21). From January to December of 2015, we measured the 25(OH)vitamin D levels of the schizophreniform and autistic patients in our specialized ward for these diseases, with the aim of analyzing the prevalence of 25(OH)vitamin D deficiency in this 1-year cohort of adult inpatients in Germany. We hypothesized that there would be increased rates of 25(OH)vitamin D deficiency in both patient groups, when compared with the large German reference cohort. Moreover, we conducted an exploratory analysis of the possible correlations between the 25(OH)vitamin D levels and psychopathological scores.

## Participants and Methods

### Study Sample

For this research, we included patients with schizophreniform and high-functioning autism spectrum syndromes, who were admitted to our specialized ward from January to December of 2015. The study was part of a larger project analyzing immunological markers, which received approval from the local ethics committee (Faculty of Medicine, Freiburg University, EK-Fr 609/14). Those patients who had been transferred from our sheltered ward to our special unit for schizophreniform and autism spectrum disorders were excluded, since they did not receive this standard diagnostic procedure. Similarly, those patients who were already being treated with vitamin D were excluded (*N* = 2). The diagnostic procedure was conducted by experienced in-house senior consultant psychiatrists, following the criteria of the International Classification of Diseases, tenth revision. This approach led to the inclusion of 60 schizophreniform and 23 high-functioning autism spectrum patients in our study. The schizophreniform syndrome group was comprised of 41 patients with schizophrenia, 7 with schizoaffective disorders, 6 with organic schizophreniform disorders, 2 with substance-induced psychosis, and 4 patients each with acute polymorphic psychotic disorder, organic hallucinations, delusional disorder, or schizotypal disorder. The autism spectrum group included 9 patients with Asperger’s syndrome and 14 with atypical autism. The autistic patients were admitted to our clinic for diagnostic purposes and participation in a specific psychotherapy program to improve social interaction (The Freiburg Asperger Specific Therapy Manual for Adult Patients or the FASTER program) (22), as well as for treatment of comorbidities (e.g., depression). All of the patients were Caucasian and lived in Germany. The historical German control cohort was collected from the Robert Koch Institute in 1998 within the scope of the German health survey, which analyzed 3,917 adult subjects between 18 and 79 years old (13).

### Laboratory Measurements

The laboratory measurements of the patients’ 25(OH)vitamin D levels were determined using a chemiluminescence immunoassay at our Institute for Clinical Chemistry and Laboratory Medicine.^3^ Vitamin D deficiency was defined as serum 25(OH)vitamin D levels <20 ng/ml, relative vitamin D insufficiency was 20–30 ng/ml, and the preferred vitamin D range was >30–60 ng/ml (1, 9, 11).^4^ The control group was also analyzed using a chemiluminescence immunoassay (13).

### Psychometric Data

The psychopathological scores for attention and memory, formal thought disorder, fear and compulsion, delusions, affectivity, energy and psychomotor domain, circadian rhythm, and suicidal tendency were acquired in a standardized way according to the AMDP system (Arbeitsgemeinschaft für Methodik und Dokumentation in der Psychiatrie^5^). Together with the sociodemographic data, all of the data were obtained using our clinic’s electronic documentation system.

### Statistical Analyses

The statistical analyses were performed using the Statistical Package for the Social Sciences software, version 22 (SPSS 22^6^) and R software, version 3.2.2.^7^ The main results were presented descriptively. The group comparisons (i.e., schizophreniform/autism patient groups vs. historical controls) for the rate of decreased 25(OH)vitamin D levels were calculated using Pearson’s two-sided chi-squared test and Yates’ continuity correction (in *R*). The correlation analyses between the 25(OH)vitamin D levels and psychometric scores were conducted separately for each group using the Pearson correlation coefficient (in SPSS 22). For the statistical analyses used to develop further hypotheses, a *p*-value <0.05 served as the criterion of significance.

## Results

### Sociodemographic Data

The average age of the entire cohort was 32.98 ± 11.05 years old, ranging from 18 to 71 years old, and male patients were predominantly included in both patient groups. The sociodemographic details are presented in Table 1. Fifty-five of the 60 schizophreniform patients and 15 of the 23 autism spectrum patients were medicated. Fifty patients of the schizophreniform group received neuroleptics; 29 schizophreniform patients received neuroleptics as a monotherapy, in 21 patients in combination (e.g., with anticonvulsants or lithium in patients with schizoaffective disorders). The autistic patients were treated with antidepressants and/or neuroleptics.

**Table 1:** **Vitamin D findings in the schizophreniform and autism spectrum syndrome groups**.

	Schizophreniform syndromes (*n* = 60)	Autism spectrum syndromes (*n* = 23)	Entire patient cohort (*n* = 83)	German control group (*n* = 3 917)^a^
**Demographic information**				
Age – *mean* ± *SD*	33.5 ± 11.3	31.7 ± 10.6	33.0 ± 11.1	n.a.^b^
Age – *range*	18–71 years	19–57 years	18–71 years	18–79 years
Gender – *ratio*	35 males:25 females	16 males:7 females	51 males:32 females	1,706 males:2,211 females
**Laboratory findings**				
Vitamin D levels (in ng/ml) – *mean* ± *SD*	15.0 ± 9.8	14.5 ± 9.8	14.9 ± 9.8	18 ± 12.6^c^
Vitamin D levels from 0 to 5 ng/ml	6 (10%)	2 (8.7%)	8 (9.6%)	2%
Vitamin D levels from 5 to 10 ng/ml	17 (28.3%)	10 (43.5%)	27 (32.5%)	14.3%
Severe vitamin D deficiency (levels <10 ng/ml)	23 (38.3%)	12 (52.2%)	35 (42.2%)	16.3%
Vitamin D levels from 10 to 20 ng/ml	25 (41.7%)	6 (26.1%)	31 (37.3%)	41%
Overall vitamin D deficiency (levels <20 ng/ml)	48 (80%)	18 (78.3%)	66 (79.5%)	57.3%
Relative insufficiency of vitamin D levels from 20 to 30 ng/ml	9 (15.0%)	3 (13.0%)	12 (14.5%)	20.8%
Recommended vitamin D levels from 30 to 60 ng/ml	3 (5.0%)	2 (8.7%)	5 (6.0%)	21.9%^d^

*^a^Reported in Linseisen et al. (13)*.

*^b^Information not available*.

*^c^SD for the historical control group was post hoc and calculated by us*.

*^d^Reported are values >30 ng/ml*.

*Abbreviation: SD, standard deviation*.

### Laboratory Findings

**Figure 1:**
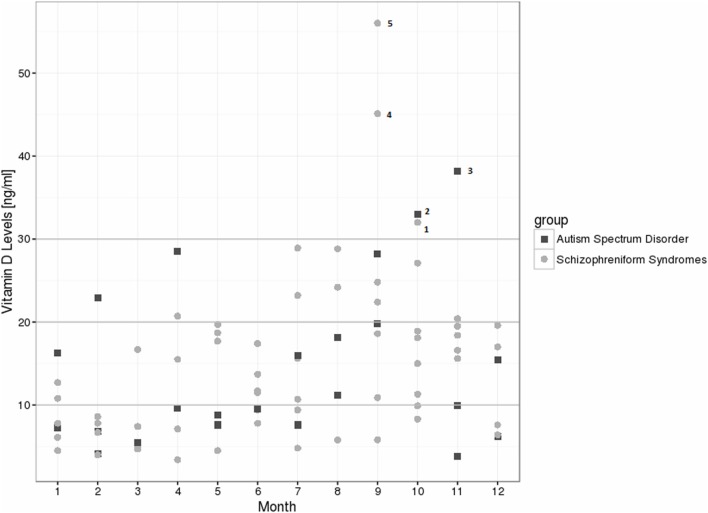
**Vitamin D level distribution over 1 year among the schizophreniform and autism spectrum disorder groups**. Five patients reached recommended levels >30 ng/ml: *patient 1*: 43 years, female, schizophreniform syndrome (organic schizophreniform disorder), treated with olanzapine, body mass index (BMI) of 26.7 kg/m^2^; *patient 2*: 25 years, male, autism spectrum disorder (Asperger syndrome), no psychiatric medication, BMI of 19.4 kg/m^2^; *patient 3*: 41 years, male, autism spectrum disorder (atypical autism), treated with olanzapine and clomipramine, BMI of 23.7 kg/m^2^; *patient 4*: 37 years, male, schizophreniform syndrome (schizophrenia), treated with olanzapine and escitalopram, BMI of 25.6 kg/m^2^; *patient 5*: 21 years, male, schizophreniform syndrome (schizoaffective disorder), treated with quetiapine and venlafaxine, BMI of 26.4 kg/m^2^.

For the schizophrenia spectrum group, we found decreased 25(OH)vitamin D levels in 80% of the patients, and relative insufficiency in 15%; therefore, the preferred 25(OH)vitamin D range was observed in only 5% of the cases. In the autism spectrum group, we found decreased levels in 78.3% of the patients, relative insufficiency in 13%, and preferred values in 8.7% [see Table 1 for the absolute values and exact distribution of the 25(OH)vitamin D levels]. In the control group, 25(OH)vitamin D deficiencies were detected in 57.3% of the patients, relative insufficiency in 20.8%, and a preferred 25(OH)vitamin D range in 21.9%. In particular, severe deficiencies (<10 ng/ml) were much more common in the schizophrenia (38.3%) and autism spectrum groups (52.2%), when compared to the controls (16.3%). From January to August of 2015, all of the patient measurements fell below the recommended threshold of 30 ng/ml (Figure 1). The details of patients within the recommended vitamin D levels are discussed in Figure 1. The 25(OH)vitamin D levels in the schizophrenia group (*N* = 41) were lower than among those patients with other psychotic syndromes (*N* = 19), although this finding was not statistically significant (13.64 ± 8.22 vs. 17.85 ± 12.39; *p* = 0.124).

### Statistical Analyses

25(OH)vitamin D deficiencies (<20 ng/ml) were significantly more common in the schizophrenia group, when compared with the historical controls (Chi^2^ = 11.559, df = 1, *p* = 0.001), and a trend in the same direction was detected in the autism spectrum group (Chi^2^ = 3.2964, df = 1, *p* = 0.069). A severe 25(OH)vitamin D deficiency (<10 ng/ml) was significantly more frequent in the schizophrenia (Chi^2^ = 19.131, df = 1, *p* ≤ 0.001) and autism spectrum patients (Chi^2^ = 18.826, df = 1, *p* ≤ 0.001). Vitamin D deficiency/severe deficiency in the control group did not significantly differ between the male and female groups, and the same was true for the schizophreniform and the autism spectrum patient groups. The correlation analyses for the schizophreniform group showed no significant correlations between their 25(OH)vitamin D levels and any psychopathological score; there were also no significant correlations when we analyzed unmedicated (*N* = 5) and medicated (*N* = 55) groups separately. However, for the autism spectrum group, we found a correlation between 25(OH)vitamin D levels and circadian rhythm (*r* = 0.458, *p* = 0.028; *N* = 23), as well as between 25(OH)vitamin D and energy levels (*r* = 0.421, *p* = 0.046; *N* = 23). These notable correlations were still significant in the medicated patient group (*N* = 15) but were not found in the unmedicated patients (*N* = 8).

## Discussion

The main findings of our study were decreased 25(OH)vitamin D levels in 80% of the schizophreniform patients and 78% of the patients with autism spectrum disorders. In particular, severe deficiencies (<10 ng/ml) were much more common in both patient groups, when compared to the healthy controls.

### Limitations

In this study, we have described a 1-year cohort of electively hospitalized adult patients in our special unit for schizophreniform and autism spectrum syndromes at a tertiary care university hospital in Freiburg, Germany. We compared our findings to a historical German control group of 3,917 adult subjects between 18 and 79 years old and used the same method to measure 25(OH)vitamin D in another laboratory (13). However, this control group was not matched for gender, age, body mass index, sunlight exposure, or seasonality. The (severe) vitamin D deficiency in the control group did not significantly differ between males and females, and the same was true in the schizophreniform (35 males:25 females) and autism spectrum (16 males:7 females) patient groups. Therefore, we do not believe that gender distribution had a decisive influence on our findings. However, we were unable to correct for the other influencing factors (age effects, body mass index, sunlight exposure, and seasonality), because this information was unavailable for the control group. Therefore, social withdrawal in the patient cohort *per se* might be responsible for the detected vitamin D deficiency. Moreover, the representative control cohort could have included subjects with unrecognized psychiatric diseases, because information about the prevalence of psychiatric comorbidity in the reference cohort was not available. The information about the control group is only available in German; we therefore have included the main information in Table 1. We analyzed a 1-year cohort to determine the possible effects of seasonality as a function of sun radiation, but our results are not comparable with those of previous studies of populations living at other latitudes and longitudes, due to the different levels of sun exposure. Since vitamin D levels depend on sun exposure and the intensity of radiation, it is mandatory to perform research in different regions. Moreover, *in vitro* studies have suggested an interaction between vitamin D levels and antipsychotic drugs (23), in that antipsychotic exposure is associated with lower vitamin D levels. Thus, our results may have been influenced by the effects of the medications taken by our patients. Clearly, the definition of the reference ranges for vitamin D is controversial; however, there is concurrence that vitamin D levels of less than 20 ng/ml are too low (1, 9, 11). We used these established cutoff values following the German Nutrition Society (see text footnote 1) and the American Institute of Medicine (see text footnote 2). As described in the Section “Introduction,” these references are based on calculations with respect to the distribution of the “vitamin D requirement curve” in the general population. Therefore, we cannot speak from normal values in the common sense (like a 95th percentile), because many of the healthy controls also suffered from vitamin D deficiency. Further research should explore this aspect.

Finally, the measurement of 25(OH)vitamin D using a chemiluminescence immunoassay is well established, and the preanalytical stability has been previously described as solid and reliable (24). In addition, our serum samples were analyzed directly after blood collection; therefore, the methodological aspects are unlikely to be responsible for the alterations we found.

### Comparison with Previous Studies

A systematic review of all available studies with schizophreniform cohorts through 2013, which consisted of a total of eight studies, found an overall prevalence rate of vitamin D deficiency of 65.3% (9), which is lower than the rate in our German cohort. The odd ratios indicated that children with vitamin D deficiency were 2.16 times more likely to develop schizophrenia (9). The largest uncontrolled study to date analyzing the prevalence rates in 324 psychotic patients comes from the United Kingdom (8), and the authors found lower vitamin D levels in 86% of the participants and mean vitamin D levels of 12.4 ng/ml ± 7.3 (in comparison with 14.97 ng/ml ± 9.83 in our schizophreniform patient group). Well in line with our findings, the authors described severe vitamin D deficiency (<10 ng/ml) in 49% of the subjects. We also found strong suppressed 25(OH)vitamin D levels (<10 ng/ml) significantly more often, when compared to the historical control group. The differences in the mean vitamin D levels could be explained by the different levels of sun exposure, seasonal effects (i.e., different distribution of the measurements over the year), and the inclusion of different ethnicities (i.e., a broader spectrum in the study by Lally and colleagues) (8). With respect to the subtype of the psychotic disorder, other earlier studies found that patients with schizophrenia displayed lower vitamin D levels when compared to those with non-schizophreniform psychoses (25). In line with that, we also detected a trend in this direction. Moreover, lowered vitamin D levels have previously been associated with negative symptoms (4, 26). However, in this study, we were unable to detect such correlations for the schizophreniform patients.

In the first meta-analysis of vitamin D levels in autistic patients, which included 11 studies, every single case–control study reported significantly reduced vitamin D levels in those patients with autism. Corresponding to this, the authors described a statistically significant reduction in the vitamin D levels in the autism group, when compared to the controls. However, no adult studies were included in the meta-analysis (10). Research that combined adolescents and adults (*N* = 40), and adults with autism spectrum disorders (*N* = 10) showed similar findings (10, 27, 28). When looking at dimensional associations in the correlation analyses of the autism spectrum disorder group, we found a significant dimensional association between the 25(OH)vitamin D and energy levels, as well as with the circadian rhythms, in that higher 25(OH)vitamin D levels were associated with a higher severity of symptoms. However, this observation does not support the hypothesis that vitamin D deficiency is a marker of symptom severity. Based on the small sample size, these findings clearly need further investigation.

### Pathophysiological Interpretation: Inflammation and Schizophrenia

One previous study found that acute psychotic patients had significantly lower vitamin D levels, when compared to psychotic patients in remission and healthy controls (29). One might therefore speculate that vitamin D levels can modulate disease activity in this subgroup of patients. Nevertheless, until now, we have not been able to determine whether vitamin D deficiency is the cause, or rather a sequela of the psychiatric disorder. Many findings support the idea that vitamin D-associated autoimmune processes may play a role in the primary prevention and pathogenesis of schizophreniform disorders. The most elaborate insight into the role of vitamin D in neuropsychiatric disorders comes from MS research. The role of vitamin D in immunomodulation has been supported by the discovery that vitamin D receptors are expressed in most immune cells, including those in the brain (30, 31). Since the 25-hydroxylase enzyme, the rate-limiting enzyme for vitamin D synthesis, is also presented in these immune cells, they are able to secrete vitamin D in both autocrine and paracrine ways. Earlier studies have shown that vitamin D is indirectly able to reduce the T cell stimulatory capacity. In addition, vitamin D has direct effects on immunoregulatory T lymphocytes by stimulating the production of Type 2 helper T cell cytokines and reducing the production of Type 1 helper T cell cytokines. Furthermore, vitamin D inhibits B cell differentiation and immunoglobulin secretion, as well as T cell and B cell proliferation (31). *Via* such different effects, vitamin D contributes to immunoregulation and may decrease inflammation and produce immunoprotective effects (10, 31). Therefore, vitamin D may also lead to reduced C-reactive protein levels (8). In accordance with these observations, vitamin D deficiency may result in the withdrawal of these anti-inflammatory effects and may therefore support mild inflammatory processes (32). In non-psychiatric patients, vitamin D was found to be present in the CSF; however, the CSF concentrations were lower than those found in the corresponding sera (33). Moreover, it is still unknown how vitamin D reaches the brain (34). We previously demonstrated a blood–brain barrier dysfunction in a subgroup of more than 20% of the schizophreniform patients (14), and one could speculate that this could lead to altered intrathecal vitamin D levels. Vitamin D also seems to play a role in control of infections, for instance, by influencing the risk for tuberculosis or Epstein–Barr virus infection (35, 36). Therefore, vitamin D might not only have direct effects on immunological processes but could also influence infectiological mechanisms, which might be the link to an infectious hypothesis of schizophrenia. Further research should analyze the association between vitamin D levels and other immunological serum and CSF markers. Furthermore, in terms of the neurochemical function, alterations in the γ-aminobutyric acid, glutamate, and dopamine metabolism were found to be associated with vitamin D metabolism (6, 34), which might be another way in which vitamin D contributes to the CNS information processing in schizophrenia or autism. However, the precise pathophysiology of the immunological processes in relation to vitamin D metabolism and different disorders remains unclear, and more research is necessary in this field.

### The Role of the Vitamin D Measurement in Basic Neuropsychiatric Diagnostics

Based on the current evidence, we are far from stating that vitamin D deficiency does play a critical role in the pathophysiology of schizophreniform disorders or autism. However, there is reasonable evidence that this might be the case, in particular, with respect to the established data on MS. More importantly, irrespective of such possible pathophysiological aspects, it is well established that low vitamin D levels do play a causal role in other systemic aspects of physical health (osteoporosis, muscle weakness, atherosclerosis, neoplastic disorders, diabetes mellitus, etc.) (1, 3). Due to our findings of 25(OH)vitamin D deficiency in about 80% of these patients, we believe that more frequent measurements of vitamin D levels in inpatients with schizophreniform and autism spectrum syndromes are justified. In order to do an overall assessment of the vitamin D status, the measurement of the serum 25-hydroxy vitamin D level is recommended, because it reflects total vitamin D from sunlight exposure, dietary intake, and conversion out of the stores in the liver and fatty tissue (1).

### Treatment of Patients with Vitamin D Deficiency

Interventional studies in which vitamin D has been administered in schizophreniform disorder patients have shown mixed results (37, 38). For example, in a Finnish birth cohort of over 9,000 individuals, vitamin D supplementation in the first year of life reduced the risk of schizophrenia by 77%. Interestingly, this finding was detected in males, but not in females (39). In a small study of a schizophreniform immigrant population, daily vitamin D supplementation (of 1,000 IU/day) did not lead to changes in their psychiatric symptoms (40). The mixed results of earlier studies in schizophreniform patient cohorts might be a consequence of non-comparable doses of vitamin D. Furthermore, small early interventional studies in infantile autism spectrum groups have provided encouraging results; however, studies in adult autism, especially blinded, randomized placebo-controlled trials are absent (41).

## Conclusion

We found a very high prevalence of 25(OH)vitamin D deficiency in adult patients with schizophreniform and autism spectrum syndromes admitted for inpatient treatment in a tertiary referral center for psychiatry and psychotherapy. It is unproven, but possible, that these findings may be associated with immunological alterations in a subgroup of these patients. However, irrespective of such speculative pathomechanisms, we suggest more frequent measurements of vitamin D levels in patients with these disorders, in light of the high prevalence of vitamin D deficiency. The individualized supplementation of patients with schizophreniform and autism spectrum disorders with vitamin D deficiencies may be considered, with regard to the possible consequences of further vitamin deficiency (osteoporosis, enhanced cardiovascular risk, etc.). Further prospective, controlled, randomized research should be conducted to assess the efficacy of vitamin D supplementation in patients with and without suppressed vitamin D levels and schizophreniform/autistic syndromes. If vitamin D treatment should turn out to be effective, one might also discuss supplementation with moderate doses without any measurement due to the high prevalence of vitamin D deficiency, because the measurement is very costly as compared to the vitamin itself.

## Author Contributions

LTvE and DE initiated the study and conducted the data analyses. DE wrote the paper. AB supervised the laboratory measurements. BF supported the statistical analyses. All of the authors were crucially involved in the theoretical discussion and performance of this study. Furthermore, all of the authors read and approved the final version of this manuscript.

## Conflict of Interest Statement

DE, RD, AB, EP, BF, SM, and AR: None; OS: Consulting and lecture fees, grant and research support from Bayer Vital GmbH, Biogen Idec, Genzyme, Merck Serono, Novartis, Sanofi-Aventis, and Teva; LTvE: Lectures, workshops, or travel grants within the last 3 years: Eli Lilly, Medice, Shire, UCB, Servier, and Cyberonics.

## Footnotes

^1^www.dge.de

^2^www.nationalacademies.org/hmd/

^3^www.uniklinik-freiburg.de/ikcul.html

^4^www.dge.de/wissenschaft/referenzwerte/vitamin-d/

^5^www.amdp.de

^6^www-01.ibm.com/software/analytics/spss

^7^www.r-project.org
